# Two Kinesin-14A Motors Oligomerize to Drive Poleward Microtubule Convergence for Acentrosomal Spindle Morphogenesis in *Arabidopsis thaliana*

**DOI:** 10.3389/fcell.2022.949345

**Published:** 2022-07-22

**Authors:** Takashi Hotta, Yuh-Ru Julie Lee, Takumi Higaki, Takashi Hashimoto, Bo Liu

**Affiliations:** ^1^ Department of Plant Biology, College of Biological Sciences, University of California, Davis, Davis, CA, United States; ^2^ Department of Cell and Developmental Biology, University of Michigan Medical School, Ann Arbor, MI, United States; ^3^ Division of Biological Science, Nara Institute of Science and Technology, Ikoma, Japan; ^4^ Faculty of Advanced Science and Technology, Kumamoto University, Kumamoto, Japan; ^5^ International Research Organization for Advanced Science and Technology, Kumamoto University, Kumamoto, Japan

**Keywords:** kinesin-14, kinetochore fibers, interpolar microtubules, microtubule convergence, mitotic spindle, spindle poles, *Arabidopsis*

## Abstract

Plant cells form acentrosomal spindles with microtubules (MTs) converged toward two structurally undefined poles by employing MT minus end-directed Kinesin-14 motors. To date, it is unclear whether the convergent bipolar MT array assumes unified poles in plant spindles, and if so, how such a goal is achieved. Among six classes of Kinesin-14 motors in *Arabidopsis thaliana*, the Kinesin-14A motors ATK1 (KatA) and ATK5 share the essential function in spindle morphogenesis. To understand how the two functionally redundant Kinesin-14A motors contributed to the spindle assembly, we had ATK1-GFP and ATK5-GFP fusion proteins expressed in their corresponding null mutants and found that they were functionally comparable to their native forms. Although ATK1 was a nuclear protein and ATK5 cytoplasmic prior to nuclear envelop breakdown, at later mitotic stages, the two motors shared similar localization patterns of uniform association with both spindle and phragmoplast MTs. We found that ATK1 and ATK5 were rapidly concentrated toward unified polar foci when cells were under hyperosmotic conditions. Concomitantly, spindle poles became perfectly focused as if there were centrosome-like MT-organizing centers where ATK1 and ATK5 were highly enriched and at which kinetochore fibers pointed. The separation of ATK1/ATK5-highlighted MTs from those of kinetochore fibers suggested that the motors translocated interpolar MTs. Our protein purification and live-cell imaging results showed that ATK1 and ATK5 are associated with each other *in vivo*. The stress-induced spindle pole convergence was also accompanied by poleward accumulation of the MT nucleator γ-tubulin. These results led to the conclusion that the two Kinesin-14A motors formed oligomeric motor complexes that drove MT translocation toward the spindle pole to establish acentrosomal spindles with convergent poles.

## Introduction

Bipolar spindles ensure equal distribution of the replicated genetic material to two daughter cells during cell division. In fungi and animal cells, spindles often have their poles defined by the spindle pole bodies and centrosomes, respectively. Plants and certain reproductive cells of centrosome-producing animals form acentrosomal spindles that are equally effective to fulfill the task of chromosome segregation (Dumont and Desai, 2012; Meunier and Vernos, 2016; Yamada and Goshima, 2017). Acentrosomal spindles have microtubules (MTs) converged toward undefined or diffuse poles that vary in their widths. Compared to the centrosome or spindle pole body as structurally defined MT-Organizing Centers (MTOCs) in animal and fungal cells, such diffuse spindle poles in plant cells represent a form of structurally undefined, pleiomorphic MTOCs (Brown and Lemmon, 2007). At acentrosomal spindle poles, MTs seem to be “glued” together by proteins that preferentially associate with their minus ends. Such a phenomenon was particularly conspicuous during mitosis in liverworts in which centrosome-like polar organizers (POs) define spindle poles (Buschmann et al., 2016). The POs are enriched with the MT nucleator γ-tubulin (Brown and Lemmon, 2006), which may be associated with a potential function in generating new MTs during mitosis. Although it is currently unclear what proteins in addition to γ-tubulin are present there, the POs may represent a transition from centrosomal to acentrosomal spindle assembly in evolution (Buschmann and Zachgo, 2016). Analogously, acentrosomal spindles produced by animals also have proteins that function in MT nucleation and act at MT minus ends concentrated at the poles (Mogessie et al., 2018).

Among the proteins that act in acentrosomal spindle pole convergence, the MT-associated protein (MAP) NuMA (nuclear and mitotic apparatus) and its associated MT motor cytoplasmic dynein often are considered to be the most prominent players for this job in animal cells (Hueschen et al., 2017). A plausible model is that the NuMA protein acts as a matrix and recruits cytoplasmic dynein to cluster MT minus ends *via* its minus end-directed motility (Radulescu and Cleveland, 2010). Another type of MT minus end-directed motors, Kinesin-14 also plays a synergistic role with cytoplasmic dynein for spindle pole focusing in animal cells (Wittmann et al., 2001; Goshima et al., 2005). Angiosperms lack both NuMA and cytoplasmic dynein but contain Kinesin-14 motors (Yamada and Goshima, 2017). Plant Kinesin-14 motors can be grouped into six classes including the Kinesin-14A class with members sharing similar structural features as the yeast Kar3p, fruit fly Ncd, and human HSET (Guo et al., 2009). Because of bearing a nucleotide-independent MT-binding domain, these Kinesin-14 motors potentially can cluster MT minus ends together so that they could serve as the primary driving force for MT convergence during spindle pole formation in the absence of cytoplasmic dynein, a scenario in plant cells.

Spindles formed in plant cells exhibit great plasticity in their morphologies, especially in regard to the width of their poles so that they often are described as barrel-shaped (Palevitz, 1993). Nevertheless, spindle MTs universally converge albeit at different degrees in different plant cells. The significance of such MT convergence is beginning to be recognized with the aid of molecular genetics. In *A*. *thaliana*, for example, the *atk1-1* mutant exhibits the phenotype of reduced fertility due to frequent failures in male meiosis due to disturbed chromosome segregation. This phenotype is caused by the inactivation of a gene encoding the Kinesin-14A motor ATK1 (or KATA) in the Kinesin-14 subfamily (Chen et al., 2002). The male sterile phenotype of *atk1-1* is linked to the formation of meiotic spindles with broadened poles and kinetochore fibers are aligned towards multiple poles in microsporocytes when compared to highly focused spindle poles in wild-type cells (Chen et al., 2002). A mutation in the maize ortholog of this motor, *dv1* (*divergent spindle 1*) also leads to a similar phenotype in male meiosis and decreased pollen viability (Higgins et al., 2016). Therefore, spindle pole focusing is a critical factor contributing to the faithful segregation of chromosomes during karyokinesis in microsporocytes. When the expression of ATK1 protein is not limited to microsporocytes, the fact that the spindle phenotype was readily detected in male meiosis but not mitosis suggests that microsporocytes respond to challenges in spindle deformation more sensitively than megasporocytes and mitotic cells. In addition, because ATK1 is a non-processive, minus end-directed motor (Marcus et al., 2002), it is unknown how it can drive MT convergence towards spindle poles.

In *A*. *thaliana*, another Kinesin-14A motor ATK5 shares greater than 80% identity in amino acid sequence and plays a redundant role in spindle assembly with ATK1, and simultaneous inactivation of both genes leads to lethality (Quan et al., 2008). Despite the functional redundancy, the *atk5* single mutant produces mitotic spindles with widened poles, suggesting that both ATK1 and ATK5 are required for spindle morphogenesis (Ambrose et al., 2005). An intriguing finding is that ATK5 acted as a MT plus end-tracking protein inside the spindle when ectopically expressed in a fluorescent protein-tagged fusion in tobacco cultured cells, which led to the hypothesis of having a motor act at MT plus ends to regulate MT minus-end organization at spindle poles (Ambrose et al., 2005). However, it is unclear whether such an action could be applied to ATK1 as well.

Understanding spindle morphogenesis in flowering plants can provide insights into general principles of acentrosomal spindle assembly in part because their cells do not produce the centrosome structure. To gain insights into how the acentrosomal spindle harnesses the incompletely redundant functions of the Kinesin-14A1/ATK1 and Kinesin-14A2/ATK5 kinesins to establish convergent spindle poles, we observed the dynamic localization of the two motors expressed under their native promoters in mitotic cells in *A*. *thaliana*. Our results revealed that they were heavily associated with selective groups of spindle MTs and could be induced to converge MTs at their minus ends to form perfectly focused, centrosome-like asters. Because the two motors physically associated with each other *in vivo*, our results supported the notion that the motors formed oligomers in order to express the spindle pole-focusing function during mitosis. For brevity and convenience, these two motors are described as ATK1 and ATK5 hereinafter.

## Materials and Methods

### Plant Materials and Growth Conditions

The *A*. *thaliana* mutants used in this study were *atk1-1* (Chen et al., 2002) provided to us by H. Ma and *atk5* (WISCDSLOX470B5/CS857307) acquired from the Arabidopsis Biological Research Center (ABRC) located at Ohio State University in Columbus, Ohio, and the *eb1a*/*b*/*c* triple mutant (Bisgrove et al., 2008). The *atk5* mutation was detected by PCR by using primers of 857307RP (5′-CAA ATA AAC GTA TGT CAG TGT AAA GAA AC-3′) and p745 (5′-AAC GTC CGC AAT GTG TTA AGT TGT C-3′), and the wild-type allele was detected by 857307LP (5′-AAA TCA CAG AAG AGA AAA AGA TTG TAG AG-3′) and 857307RP. All plants were grown in soil with an illumination cycle of 16-h light and 8-h dark at 70% relative humidity at 22°C in growth chambers located in the College of Biological Sciences at University of California in Davis. For root microscopic imaging purposes, seedlings were produced on a solid medium supplied with ½ Murashige Skoog (MS) salt mixture and 0.8% Phytagel (Sigma).

Tobacco (*N*. *bethamiana*) plants were grown in a growth chamber under the 16-h light and 8-h dark cycle at 25°C, as described previously (Xu et al., 2020).

### Construction of Expression Vectors

The *ATK1* and *ATK5* genes correspond to the gene models of AT4G21270 and AT4G05190, respectively, in the *A*. *thaliana* genome. To produce an ATK1-GFP construct, a 4864-bp genomic fragment including a 561-bp hypothesized promoter region, was amplified using the primers ATK1Fw (5′-CAC CTT TGT TTT TCT TTC TCA AGA CGA AAA TTG AAG C-3′) and ATK1-Rv (5′- GCC ATA GCT TAA GCG AGA GTC AAG GAG CTT C-3′) using the Phusion DNA polymerase (Thermo Fisher). The fragment was inserted into the pENTR-D/TOPO vector (Thermo Fisher), according to the manufacturer’s instruction to generate the pENTR-ATK1 plasmid. The pENTR-ATK5 plasmid was produced after the amplification of a 6175-bp genomic fragment including a 2,035-bp promoter region by using the primers of IV05190FB1 (5′-GGG GAC AAG TTT GTA CAA AAA AGC AGG CTA ATA TGT TGA CAC GTG GTT TCT TGA-3′) and IV05190R_B2 (5′-GGG GAC CAC TTT GTA CAA GAA AGC TGG GTC ACC GTA ACT TAG GCG AGA GTC GAG-3′) and cloning into the pDONR221 *via* recombination by BP clonase (Thermo Fisher). The ATK1-GFP and ATK5-GFP expression vectors were produced by LR recombination reactions (Thermo Fisher) between the pGWB4 plasmid (Nakagawa et al., 2007) and the pENTR-ATK1 and pENTR-ATK5 plasmids, respectively.

To mark MTs when ATK1, ATK5, and other proteins were tagged with GFP, a mCherry-TUB6 (β-tubulin 6) construct, as reported previously (Liu et al., 2018), was used for stable transformation and transient expression experiments.

To reconstitute superfolder (sf) GFP from sfGFP^1-10^ and sfGFP^11x7^, corresponding DNA fragments, as published (Kamiyama et al., 2016) were synthesized in gBlocks (Integrated DNA Technologies) and cloned into pGWB601 and pGWB616 (Nakamura et al., 2010) by Gibson Assembly reactions (New England Biological) to give rise to pLB128 and pFP7, respectively. The ATK1-sfGFP^1-10^, ATK5-sfGFP^11x7^, and AUG3-sfGFP^1-10^ constructs were produced by LR reactions between pENTR-ATK1 and pLB128, pENTR-ATK5 and pFP7, and pENTR-AUG3 (Ho et al., 2011) and pLB128, respectively.

### Leaf Infiltration and Transformation Experiments

All expression constructs were transformed into competent cells of the *Agrobacterium tumefacien* strain GV3101 prior to transient and stable expressions. Transient expression was carried out by infiltrating tobacco leaves with bacterial suspensions, as described previously (Xu et al., 2020). Stable transformation was carried out by adopting the floral dip protocol in *A*. *thaliana* (Clough and Bent, 1998).

### Confocal and Immunofluorescence Microscopy

For the observation of mitosis in root cells, 4-day-old *A*. *thaliana* seedlings were mounted under a coverslip with water. To observe induced mitosis in tobacco epidermal cells, we had leaf segments excised 48 h after infiltration and mitotic induction by the expression of cyclin D3; 1 (Xu et al., 2020) and mounted under a coverslip with water. All live-cell imaging experiments were performed under an LSM710 laser scanning confocal module mounted on an Axio Observer inverted microscope by using a 40X water immersion objective (Carl Zeiss).

The osmotic stress was brought about by treating roots with 0.3 M sorbitol solution for 30 min prior to fixation by paraformaldehyde for immunofluorescence. Live-cell imaging of the stressed roots was carried out by having the sample exposed to an agar block containing sorbitol at an identical concentration under a confocal microscope.

To detect proteins in isolated meristematic cells, roots were excised from germinated seedlings and fixed by paraformaldehyde and processed for immunolocalization experiments as described previously (Hotta et al., 2012). The DM1A anti-α-tubulin monoclonal antibody (Millipore Sigma) was used to detect MTs, and affinity-purified polyclonal anti-GFP antibodies were used to probe GFP-tagged proteins (Kong et al., 2010). These primary antibodies were detected by fluorescein isothiocyanate (FITC)-conjugated donkey anti-rabbit IgG and Texas Red-conjugated donkey anti-mouse IgG antibodies (Rockland), respectively, while DNA was labeled by the fluorescent dye DAPI at 1 μg/ml. Cells were observed under an Eclipse 600 epifluorescence microscope with a Plan-Fluor 100x objective (Nikon), and images were acquired by an OptiMOS sCMOS camera (Photometrics).

To merge acquired images for presentation, pseudo-colors were assigned to the images before being merged in Metamorph software (Molecular Devices) and the ImageJ program. Figure plates were assembled in Adobe Photoshop (Adobe).

### Immunoaffinity Purification and Protein Identification

Proteins were extracted from the transgenic ATK1-GFP and ATK5-GFP plants, respectively, prior to being applied to the column of anti-GFP magnetic beads using the µMACS™ GFP Isolation Kit (Miltenyi Biotec) as described previously (Lee et al., 2017). Proteins were eluted from the column by using SDS-PAGE sample buffer followed by electrophoresis. The purified protein samples were submitted to the Taplin Mass Spectrometry Facility at Harvard Medical School for mass spectrometric analysis followed by searching against the Arabidopsis proteome assembled in the TAIR10 genome database at the Arabidopsis Information Resource (TAIR).

## Results

In *A*. *thaliana*, many kinesin genes like *ATK1* and *ATK5* exhibit a cell cycle-dependent expression manner (Vanstraelen et al., 2006); therefore, such motors are expected to have spatiotemporally regulated functions. To capture their activities in mitotic cells of their origin, we had ATK1-GFP and ATK5-GFP fusion proteins expressed under the control of their native promoters and their functionality verified prior to *in vivo* imaging.

### Establishment of Functional ATK1-GFP and ATK5-GFP Lines

**FIGURE 1:**
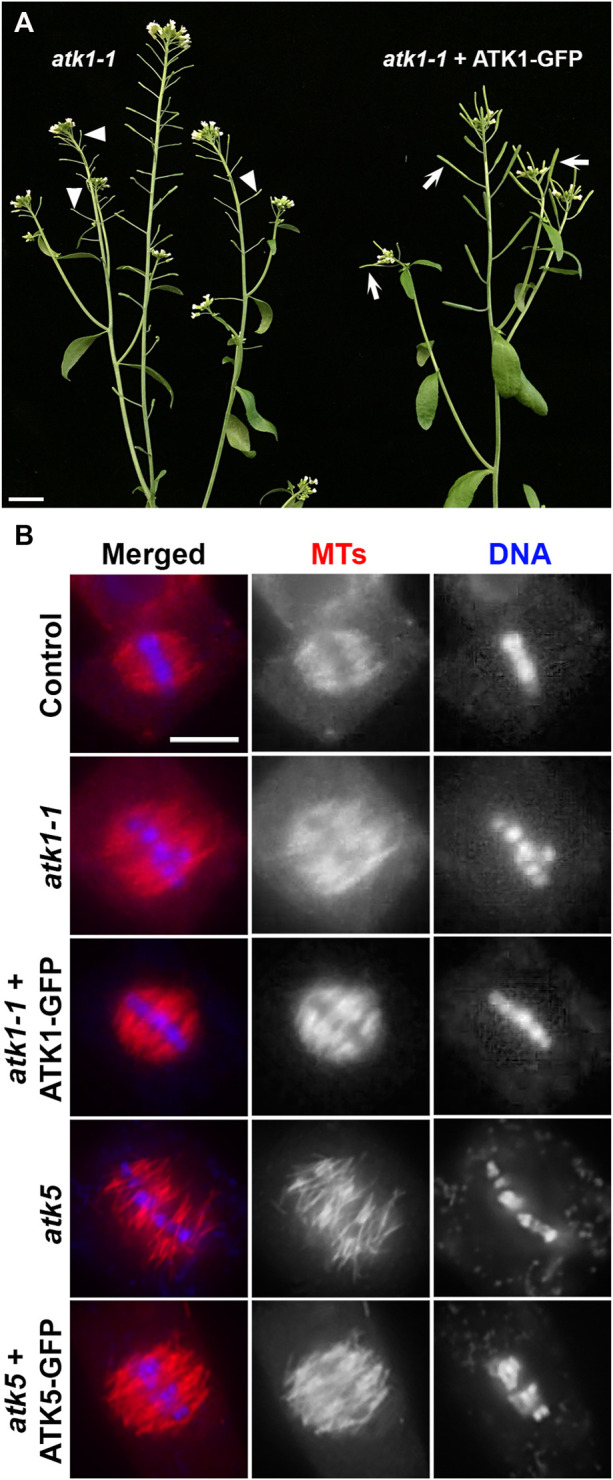
GFP fusion proteins of ATK1 and ATK5 function in spindle MT organization in *A*. *thaliana*. **(A)** Male sterile phenotype, reflected by unelongated siliques (arrowheads), is suppressed by the expression of the ATK1-GFP fusion protein that resulted in the production of seed-filled siliques (arrows). **(B)** Spindle MT organization by anti-tubulin immunofluorescence in the *atk1-1* mutant and the mutant expressing ATK1-GFP, *atk5,* and the *atk5* mutant expressing the ATK5-GFP fusion. Both *atk1-1* and *atk5* mutants produced metaphase spindles that do not show obvious spindle poles and lack poleward MT convergence. Similar to the wild-type control, the expressions of ATK1-GFP and ATK5-GFP, respectively, restored the fusiform spindle morphology. Scale bars, 1 cm **(A)** and 5 µm **(B)**.

To examine the dynamics of the two Kinesin-14A motors during mitosis, we aimed to have ATK1-GFP and ATK5-GFP fusion proteins expressed under the control of their native promoters. We first used the *atk1-1* mutant to test the functionality of the ATK1-GFP fusion protein. As reported, the *atk1-1* mutation caused male sterility due to meiotic defects that resulted in unelongated siliques that lacked seeds (Chen et al., 2002), the expression of the ATK1-GFP fusion brought the fertility back as testified by fully elongated siliques filled with seeds (Figure 1A). Therefore, we concluded that the C-terminal GFP tagging of ATK1 and similar motors would not noticeably affect the *in vivo* function. To confirm this notion, we examined spindle MT arrays in the *atk1-1* mutant and the plant expressing ATK1-GFP in comparison to the wild-type control. The expression of ATK1-GFP had spindle morphology restored into a fusiform-shaped apparatus, as seen in the wild-type control, from ones having defective spindle pole organization (Figure 1B).

Similarly, we had an ATK5-GFP expressed in the *atk5* mutant and examined spindle morphology because there were no macroscopic and seedling growth phenotypes linked to the *atk5* null mutant (Ambrose et al., 2005). Upon the expression of ATK5-GFP, the defective spindle phenotype in the *atk5* mutant was suppressed and the convergent spindle MT array was indistinguishable from that in the wild-type control (Figure 1B).

### ATK1 and ATK5 Act on the Spindle MTs at Different Times

**FIGURE 2:**
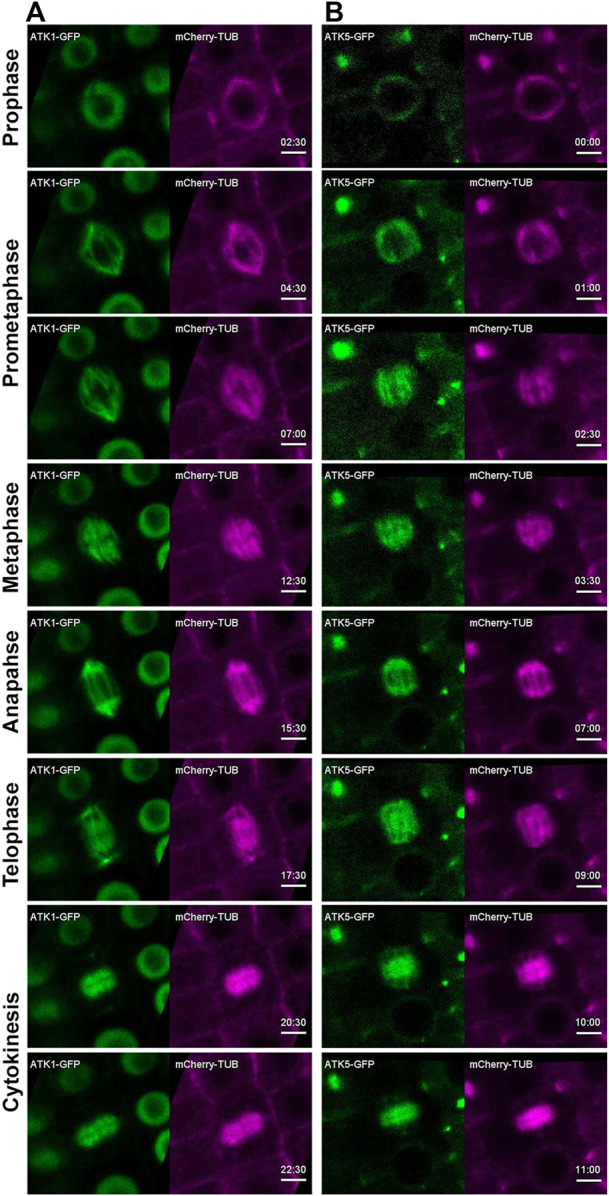
ATK1-GFP and ATK5-GFP associate with spindle MTs in *A*. *thaliana*. Time stamps are in min: sec. **(A)** Snapshots of time-lapse movie of a mitotic cell expressing ATK1-GFP and mCherry-TUB6 (for MTs). ATK1-GFP is a nuclear protein prior to nuclear envelope breakdown (2:30). After prophase, the ATK1-GFP signal largely overlaps with that of MTs in the spindle apparatus at prometaphase (4:30 and 7:00), metaphase (7:30), and anaphase (15:30). ATK1-GFP and MTs also colocalize in the developing phragmoplast at telophase (17:30) and cytokinesis (20:30 and 22:30). **(B)** In contrast to ATK1 as a nuclear protein prior to nuclear envelope breakdown, ATK5-GFP continuously decorates mitotic MT arrays uniformly from prophase (00:00) to prometaphase (1:00 and 2:30), metaphase (3:30), and anaphase (7:00). ATK5-GFP also decorates the phragmoplast MT array (9:00-11:00). Scale bars, 5 µm.

Using the ATK1-GFP and ATK5-GFP lines described above, we examined their dynamic localization during mitosis in living cells that co-expressed a mCherry-TUB6 fusion protein that was expressed under the control of native TUB6 promoter and marked all MT arrays in both interphase and mitotic cells (Liu et al., 2018). Prior to nuclear envelope breakdown at prophase when a bipolar MT array was established as a prophase spindle, ATK1-GFP resided in the nucleus but avoided the nucleolus (Figure 2A, Supplementary Movie S1). Concomitantly to the nuclear envelope breakdown, ATK1-GFP became associated with spindle and later phragmoplast MTs, and the two signals of ATK1-GFP and mCherry-TUB6 largely overlapped and were indistinguishable (Figure 2A, Supplementary Movie S1). The results suggested that ATK1 might have a nonselective association with spindle and phragmoplast MT arrays after nuclear envelope breakdown during mitotic cell division.

In contrast to ATK1, ATK5-GFP was already detected on MTs of the preprophase band and prophase spindle, and it decorated the MT array and was undetectable in the nucleus (Figure 2B, Supplementary Movie S2). Following nuclear envelope breakdown, ATK5-GFP continuously exhibited a localization pattern similar to that of MTs of spindle and phragmoplast throughout mitosis and cytokinesis (Figure 2B, Supplementary Movie S2).

### Hyperosmotic Conditions Enhance Spindle Pole Focusing

**FIGURE 3:**
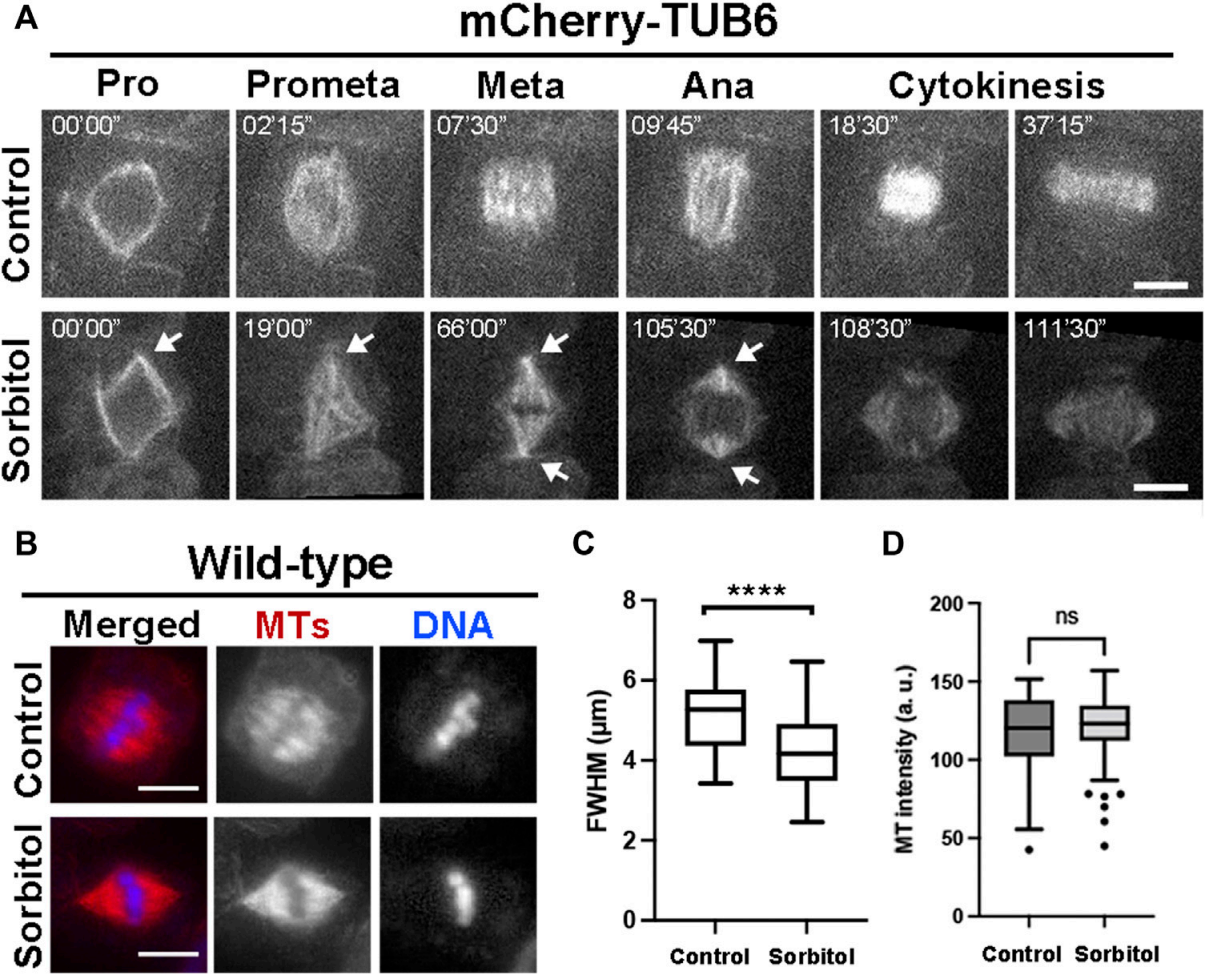
Induction of highly converged spindle poles under hyperosmotic conditions in *A*. *thaliana*. **(A)** Mitotic MT arrays were reported by a mCherry-TUB6 fusion protein. Without 0.3 M sorbitol treatment (control), a mitotic cell organizes MTs to prophase (Pro) spindle on the nuclear envelope. Following nuclear envelope breakdown, the mitotic spindle has MTs organized with wide spindle poles at both metaphase (Meta) and anaphase (Ana). With the sorbitol challenge, spindle MT arrays remain sharply focused with sharp poles (arrows) and MTs continuously reorganize into the phragmoplast array. **(B)** Spindle MT arrays in wild-type cells exposed to water (control) or treated with 0.3 M sorbitol, followed by anti-tubulin immunofluorescence. After sorbitol treatment, the spindle MT array with wide poles in the control is replaced by a spindle with sharply focused poles. **(C)** Quantitative assessment of spindle pole convergence by measuring the full width at the half maximum (FWHM) values of the spindles under the two conditions with statistical significance indicated. The FWHM values drop significantly after sorbitol treatment. **(D)** Quantitative assessment of MT density in metaphase spindles, following sorbitol treatment when compared to untreated cells (control). No significant (ns) difference was found. Scale bars, 5 µm.

Our previous work showed that in cultured tobacco cells MT exhibited increased degrees of convergence towards spindle poles after the cells were treated with glycerol (Liu et al., 1996). We tested whether such a phenomenon could be recapitulated in meristematic cells under hyperosmotic conditions in *A*. *thaliana*. We monitored mitotic MT arrays using control plants expressing mCherry-TUB6 under the control of the *ATML1* promoter (Wong and Hashimoto, 2017). Without osmotic challenges, mitotic cells produced spindle MT arrays with two wide poles due to different degrees of MT convergence (Figure 3A, Supplementary Movie S3). Upon the completion of prophase indicated by nuclear envelope breakdown, the mitotic cell spent nearly 30–40 min to arrive at the stage when the phragmoplast MT array was fully expanded to reach the parental plasma membrane (Figure 3A). We applied hyperosmotic conditions by exposing the seedlings to 0.3 M sorbitol, which resulted in having the greatest impact on spindle pole focusing. The prophase spindle had nicely focused poles, similar to or maybe more consolidated than those in the control cells (Figure 3A). The striking impact on spindle pole focus was revealed after nuclear envelope breakdown as the spindle poles continuously became highly focused (Figure 3A, Supplementary Movie S4). The mitotic cells often spent a long time of over 30 min at pro/metaphase as judged by the spindle appearance but were not arrested because anaphase eventually took place around 100 min after nuclear envelope breakdown. The stressed cells completed cytokinesis after anaphase onset (Figure 3A).

To quantitatively capture the altered spindle morphology upon sorbitol treatment, we assayed metaphase spindles by immunofluorescence (Figure 3B), followed by measuring the total fluorescence intensity of spindle MTs from the pole to the metaphase plate and determining the full width at half maximum (FWHM) (Supplementary Figure S2). Following the sorbitol treatment, the FWHM value dropped significantly (Figure 3C). Therefore, the sorbitol-induced morphological changes in metaphase spindles were significant, confirming that spindle poles became more focused than in the control cells.

### Poleward Accumulation of ATK1 and ATK5 Induced by Sorbitol Treatment

**FIGURE 4:**
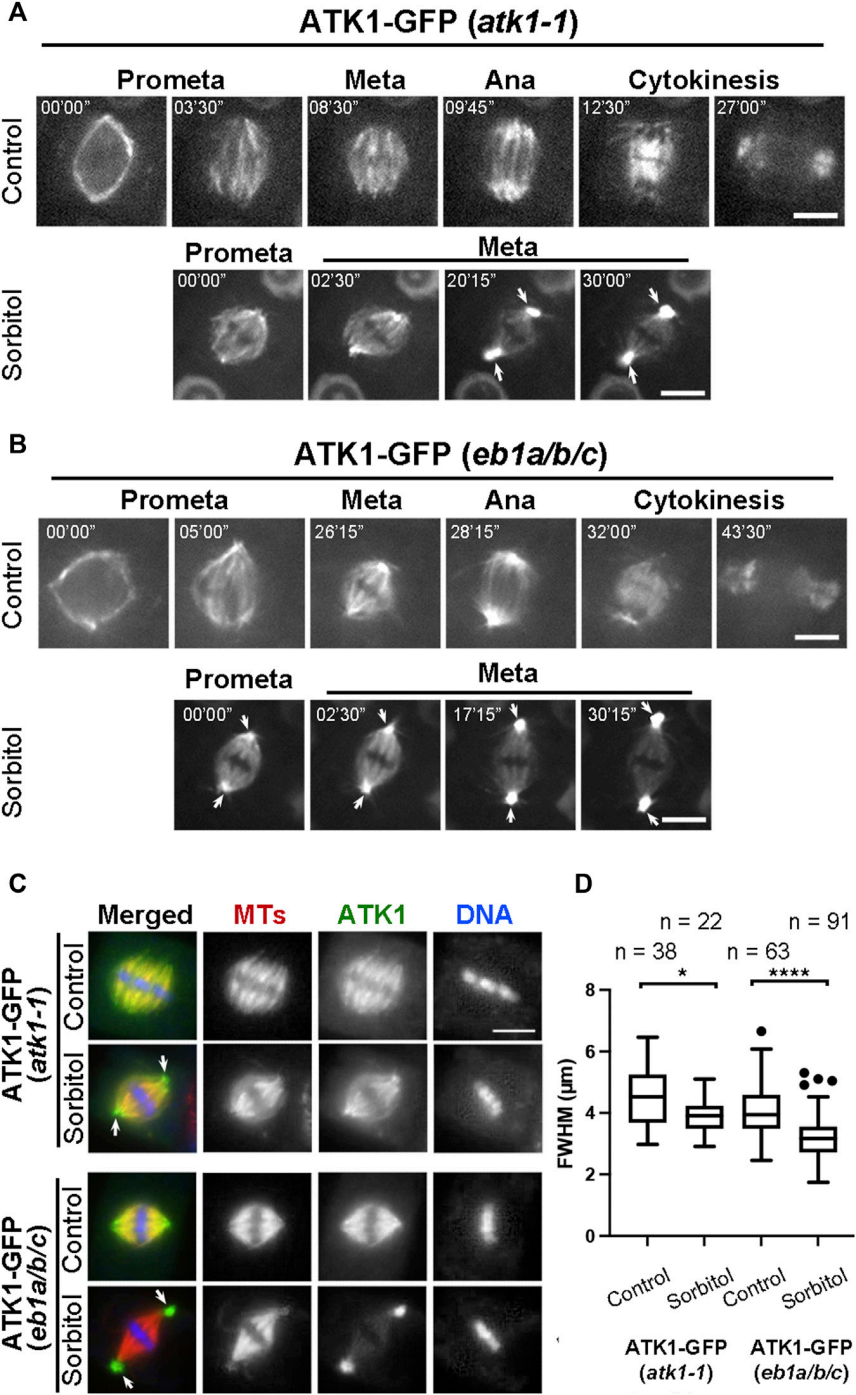
Poleward accumulation of ATK1-GFP upon sorbitol treatment in mitotic cells of *A*. *thaliana*. **(A)** In *atk1-1* cells expressing ATK1-GFP, sorbitol treatment induces redistribution of the GFP signal toward spindle poles (arrows), in contrast to the uniform signal along spindle MTs from prometaphase (Prometa) to cytokinesis without sorbitol. **(B)** Poleward ATK1-GFP accumulation is enhanced in the *eb1a/b/c* triple mutant. Without sorbitol, ATK1-GFP exhibits biased localization toward spindle poles when compared to the control cell in **(A)**. GFP signal becomes highly enriched at the poles (arrows) after sorbitol treatment while that in the spindle proper becomes diminished when the cell shows prolonged metaphase. At later stages, ATK1-GFP appears in pseudo-centrosomes at two spindle poles. **(C)** Comparison of poleward ATK1-GFP accumulation by immunofluorescence of both ATK1-GFP and MTs in the *atk1-1* rescued plant and the *eb1a/b/c* mutant background. In the rescued *atk1-1* cells, the uniform localization of ATK1-GFP along spindle MTs is replaced by a spindle pole-biased localization pattern of ATK1-GFP after sorbitol treatment (arrows). In the *eb1a/b/c* mutant background, ATK1-GFP becomes more concentrated toward the poles when compared to the uniform MT signal. The sorbitol treatment leads to the formation of ATK1-GFP-concentrated pseudo-centrosomes (arrows) that are not enriched with MTs. The spindle proper as marked by the kinetochore MT fibers has greatly diminished the ATK1 signal. **(D)** Quantitative assessment of spindle pole convergence by measuring the full width at the half maximum (FWHM) values of the spindle MT arrays. The difference between the ones by mock (water) and sorbitol treatments is greatly exaggerated in the *eb1a/b/c* mutant background. Sample numbers (n) are shown on the top. Scale bars, 5 µm.

Because sorbitol treatment induced spindle pole focusing, we asked whether such an action took place concomitantly with redistribution of Kinesin-14A motors. Compared to the uniform association of ATK1-GFP with spindle MTs prior to sorbitol treatment, we found that its signal became biased at spindle poles at prometaphase while decorating spindle MTs (Figure 4A). Later, such a pattern was followed by the accumulation of the GFP signal at spindle poles was more pronounced and ATK1-GFP often appeared in consolidated foci at spindle poles, referred to as “pseudo-centrosomes” here (Figure 4A). In the meantime, the ATK1-GFP signal on spindle MTs was gradually weakened, indicating that there was a redistribution or translocation of the signal from spindle MTs to the pseudo-centrosome.

Because the MT plus end-tracking protein EB1 marks MT polymerization away from spindle poles (Bisgrove et al., 2008), we employed an *eb1a/b/c* triple mutant in which functions of all three EB1 genes were compromised in order to find whether the mutations would enhance ATK1 accumulation at spindle poles. Prior to sorbitol treatment, ATK1-GFP appeared on spindle MTs that converged into focused poles in *eb1* triple mutant cells (Figure 4B). Upon sorbitol treatment, however, ATK1 appeared strongly at spindle poles when compared to the signal on kinetochore fibers (Figure 4B). Under this hyperosmotic condition, the signal at spindle poles became enhanced over time, concomitant with the gradual depletion of the signal along kinetochore fibers. Around 30 min after the treatment, the two pseudo-centrosome foci at opposite poles continued to exhibit outwards displacement as if they formed separated anchoring points for the spindle proper (Figure 4B). Consequently, the spindle MTs were perfectly focused on these pseudo-centrosomes that were physically separated from kinetochore fibers. The non-kinetochore fiber MTs placed between two spindle poles were taken here as interpolar MTs, the term typically used for describing MTs running between two centrosomes in animal cells. This result also suggested that ATK1 probably acted on interpolar MTs instead of kinetochore fiber MTs.

To quantify the differences in spindle morphology under these different conditions, we again measured the FWHM values of spindles in different genetic backgrounds and under different osmotic conditions. In the *atk1-1* plant expressing ATK1-GFP, mitotic cells formed spindles with relatively relaxed spindle poles so that their FWHM values were averaged greater than 4 µm. The average FWHM value dropped below 4 µm after sorbitol treatment (Figures 4C,D). In the *eb1c* triple mutant expressing ATK1-GFP; however, the average FWHM value was already below 4 µm prior to osmotic challenges, and dropped significantly to approximately 3 µm (Figures 4C,D). Therefore, we concluded that the ATK1-dependent spindle MT convergence and pole focusing was first enhanced by hyperosmotic treatments and secondly by the loss of EB1 proteins.

**FIGURE 5:**
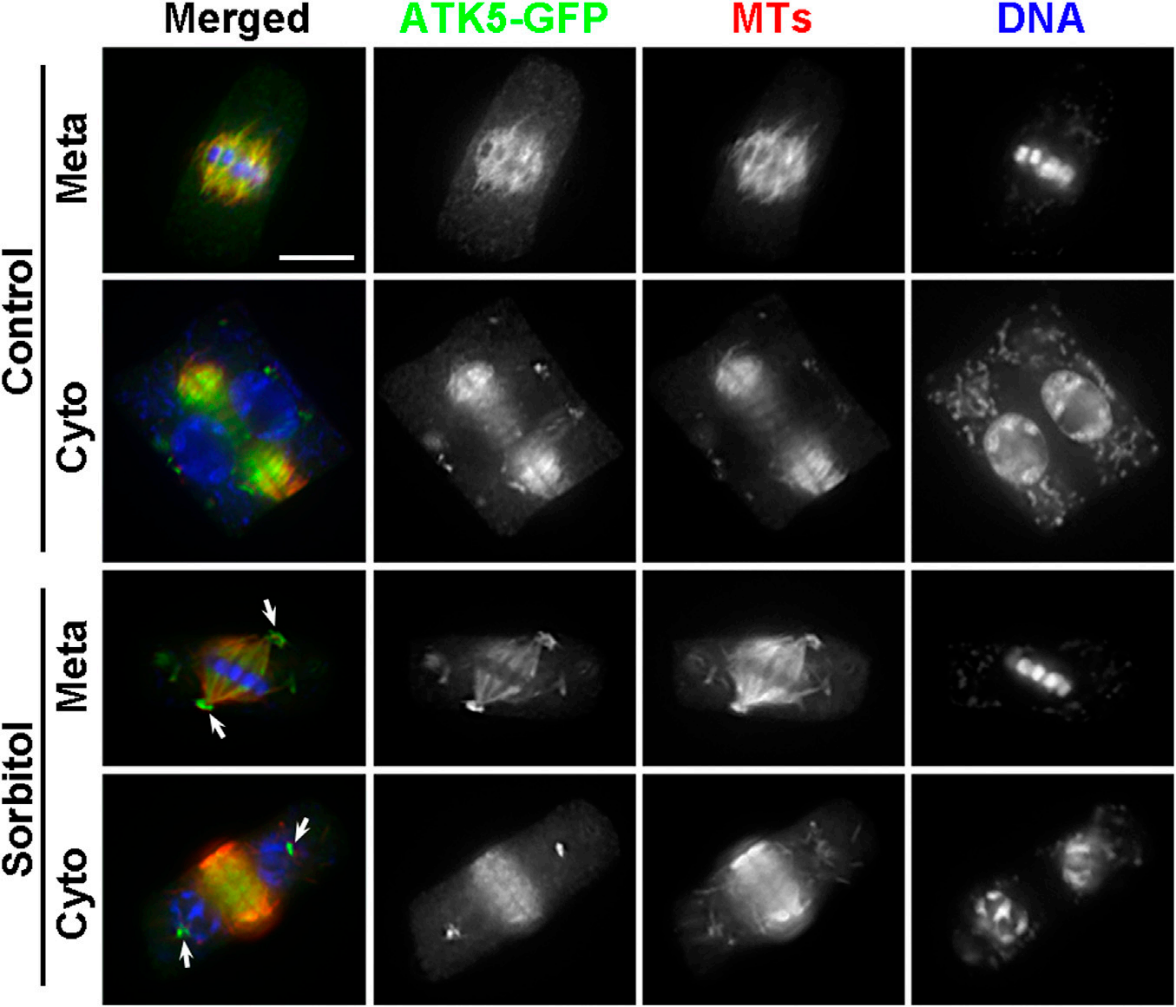
Poleward accumulation of ATK5-GFP upon sorbitol treatment in *A*. *thaliana*. The ATK5-GFP signal largely overlaps with that of MTs in both the spindle and phragmoplast arrays. Sorbitol treatment leads to the formation of the pseudo-centrosome foci in a metaphase (Meta) cell (arrows) with the signal on spindle MTs diminishing. The pseudo-centrosomes persist in the cytokinetic cell (Cyto) and appear in sharp foci (arrows) at which MTs are not concentrated. Scale bar, 5 µm.

Because ATK5 exhibited a similar localization pattern on spindle MTs, we asked whether it also exhibited poleward redistribution upon hyperosmotic treatment. When the cells expressing ATK5-GFP were treated with sorbitol, similarly, the signal became highly enriched in the pseudo-centrosome while that on spindle MTs was greatly depleted (Figure 5). The ATK5-highlighted pseudo-centrosomes persisted even when the spindle MT array was replaced by the phragmoplast array at cytokinesis (arrows, Figure 5). Therefore, we concluded that ATK1 and ATK5 had coordinated activities of poleward redistribution upon sorbitol treatment.

### Poleward Accumulation of γ-TuRC Under Hyperosmotic Conditions

**FIGURE 6:**
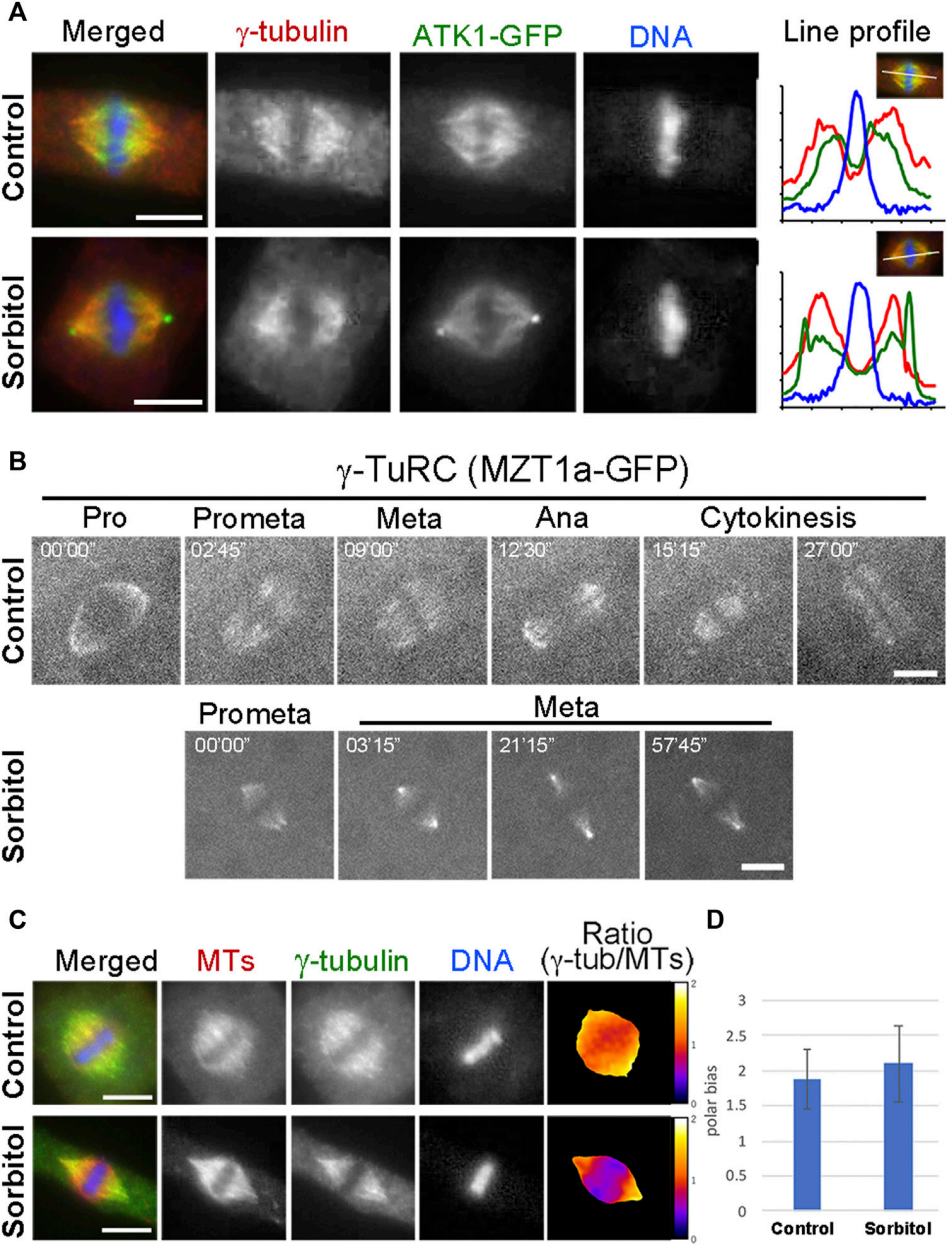
γ-TuRC exhibits a different poleward localization pattern than ATK1-GFP. **(A)** γ-Tubulin shows a spindle pole-biased localization pattern without sorbitol. But the ATK1-GFP-highlighted pseudo-centrosomes lack the γ-tubulin signal, as indicated by the line profiles reflecting the fluorescence intensities of the three signals shown. **(B)** Live-cell imaging of γ-TuRC was reported by the MZT1a-GFP fusion protein. Although there is more MZT1a-GFP signal at the two poles at later times, it never appears in discrete foci. **(C)** Comparison of γ-tubulin distribution along the spindle axis. The γ-tubulin fluorescence signal is analyzed using that of MTs as a reference. The ratio of γ-tubulin and MT signals is represented in pseudo colors with meters attached on the right. **(D)** Polar-biased localization of γ-tubulin is quantified in the control and sorbitol-treated cells. Scale bars, 5 µm.

Many MT nucleating factors like γ-tubulin and augmin exhibit spindle pole biased localization in mitotic cells (Kimmy Ho et al., 2011). Because of the sorbitol treatment-induced ATK1 accumulation at the poles, we examined whether γ-tubulin and its associated factors also exhibited a similar redistribution pattern and whether the pseudo-centrosomes were enriched with the γ-TuRC. We particularly wanted to test this possibility because γ-TuRC exhibits a predominant localization pattern at the centrosome in cultured animal cells. First, we examined γ-tubulin localization in the ATK1-GFP transgenic plants before and after sorbitol treatment. Under normal conditions, γ-tubulin exhibited a pole-biased localization pattern along spindle MTs, which was noticeable when compared to the ATK1 localization (Figure 6A). Following sorbitol treatment, the γ-tubulin signal became even more biased towards the spindle poles. But compared to γ-tubulin, ATK1-GFP became an even higher accumulation pattern at poles with the formation of the pseudo-centrosomes (Figure 6A). Surprisingly, γ-tubulin was not detected in the pseudo-centrosomes (Figure 6A). To further monitor the redistribution of the γ-TuRC following sorbitol treatment, we employed the MZT1 (mitotic spindle organizing protein 1)-GFP transgenic plant in which the γ-TuRC is highlighted by this integral factor (Nakamura et al., 2012). Compared to the typical spindle MT association of MZT1-GFP prior to sorbitol treatment, the signal became highly enriched at focused spindle poles while the cell paused at metaphase following sorbitol treatment (Figure 6B). Again, such enriched MZT1-GFP signal did not render the appearance at the pseudo-centrosome position.

Because sorbitol treatment-induced translocation of a subpopulation of spindle MTs, most likely the interpolar MTs, we asked whether γ-tubulin redistributed with these MTs. To do so, we carried out dual localizations of γ-tubulin and MTs and quantified the redistribution pattern using MTs as a reference. We found that γ-tubulin was associated with the bulk of spindle MTs and kinetochore fibers (Figure 6C). The polar biases of γ-tubulin when compared to spindle MTs, however, increased upon the sorbitol treatment (Figures 6C,D).

### ATK1 and ATK5 Physically Associate With Each Other *In Vivo*

Because ATK1 and ATK5 exhibited overlapping localization and shared function in spindle morphogenesis, we hypothesized that the two motors physically associated with each other to accomplish MT sliding activities required for MT convergence toward spindle poles. To test this hypothesis, we first used the ATK1-GFP transgenic line to purify the fusion protein by anti-GFP affinity chromatography as described previously (Lee et al., 2017). Purified proteins were subjected to mass spectrometric analysis followed by protein identification. There were 1,632 ATK1 peptides detected that covered over 85% of the ATK1 polypeptide (Table 1). To verify the specificity of the purification, we used a γ-tubulin complex protein 2 (GCP2)-GFP purification (Miao et al., 2019) as a reference, and found that neither ATK1 nor ATK5 was detected (Table 1). ATK5, however, was co-purified with ATK1-GFP (Table 1), suggesting that the two motors likely were associated with each other *in vivo*, as greater than 36% of the ATK5 polypeptide was covered after ATK1-GFP purification.

**TABLE 1:** Recovery of associated polypeptide by immunoaffinity purification of ATK1-GFP and ATK5-GFP expressed in *A*. *thaliana*. It should be noted that a GCP2-GFP purification experiment performed previously (Miao et al., 2019) was used as a negative control here. By using ATK1-GFP, ATK5-GFP, and GCP2-GFP as baits, recovered unique and total peptides and polypeptide coverage are summarized here. Detected peptides of the GFP tag provide comparisons of bait protein recovery in three purification attempts.

	Detected proteins (unique/total peptides; protein coverage)			
**Bait**	**ATK1**	**ATK5**	**GCP2**	**GFP**
ATK1-GFP	112/1,632; 85.88%	34/359; 36.20%	0; 0	21/317; 82.77%
ATK5-GFP	28/71; 38.34%	73/266; 73.42%	0; 0	11/40; 53.78%
GCP2-GFP	0; 0	0; 0	36/243; 59.20%	9/68; 58.82%

To verify this *in vivo* association, we did a reciprocal purification of ATK5-GFP, as was carried out for ATK1-GFP. The yield (266 peptides) and coverage (73%) of ATK5 were lower when compared to those of the ATK1-GFP purification, and so were those of the GFP tag (Table 1), which suggested perhaps ATK5 was less abundant than ATK1. However, ATK1 was co-purified with ATK5-GFP and had greater than 38% of the polypeptide covered, further strengthening the notion of an *in vivo* association of the two motors. Collectively, both ATK1 and ATK5 purifications did not result in the enrichment of proteins in the γ-TuRC, suggesting that the poleward accumulation of the MT nucleator was probably not a result of direct association between the nucleator and motors.

**FIGURE 7:**
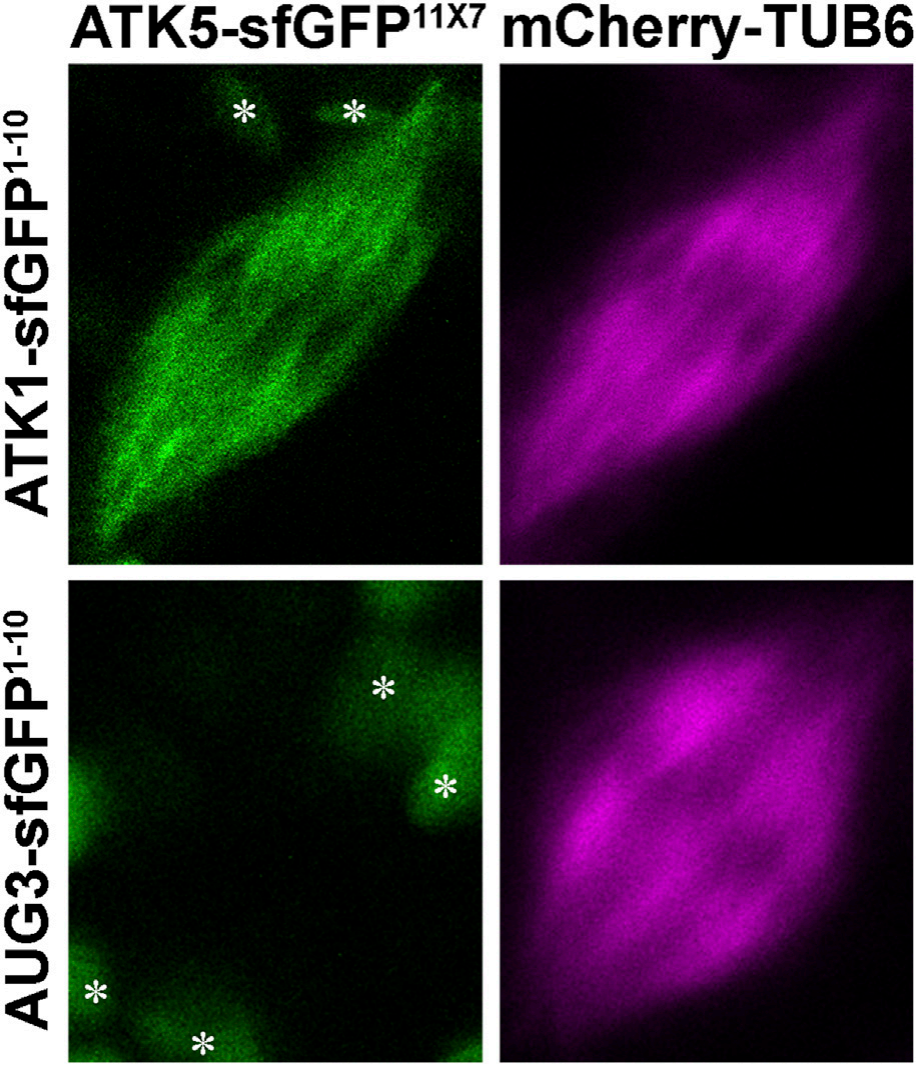
Association of ATK1 and ATK5 *in vivo* in *N*. *benthamiana* leaf cells. When leaf cells are induced to enter mitosis with spindle MT arrays marked by mCherry-TUB6, the simultaneous expressions of ATK1-sfGFP^1-10^ and ATK5-sfGFP^11X7^ result in the reconstitution of the GFP signal. When AUG3-sfGFP^1-10^ and ATK5-sfGFP^11X7^ are co-expressed, however, no GFP signal is detected on spindle MTs. The objects (asterisks) in the background are plastids with autofluorescence.

To detect such an association in living cells, we applied the split GFP technique, as described in an earlier study (Kamiyama et al., 2016). To facilitate the flexibility of the truncated sfGFP fragments, seven copies of the 11th β strand of sfGFP were made in tandem. To minimize the potential false-positive reconstitution of sfGFP, the ATK1-sfGFP^1-10^ and ATK5-sfGFP^11x7^ fusion proteins were expressed under their native promoters in order to avoid overexpression. Their co-expression in tobacco cells resulted in a fluorescent signal along spindle MTs marked by mCherry-TUB6 (Figure 7), similar to the spindle localization of ATK1-GFP and ATK5-GFP observed earlier in *A*. *thaliana* root cells. To confirm that the reconstitution of the fluorescent protein was due to the direct association of ATK1 and ATK5 but not the autonomous association of the two GFP fragments, we use the augmin subunit AUG3 as a negative control, because augmin was abundantly detected along spindle MTs (Hotta et al., 2012). When AUG3-sfGFP^1-10^ replaced ATK1-sfGFP^1-10^ and co-expressed with ATK5-sfGFP^11x7^, we never detected a GFP signal on spindles (Figure 7). Therefore, we concluded that ATK1 and ATK5 formed oligomers on spindle MTs during mitosis.

### ATK1 and ATK5 Do Not Require Each Other for Spindle MT Association but ATK1 Provides the Driving Force for Pseudo-Centrosome Formation

**FIGURE 8:**
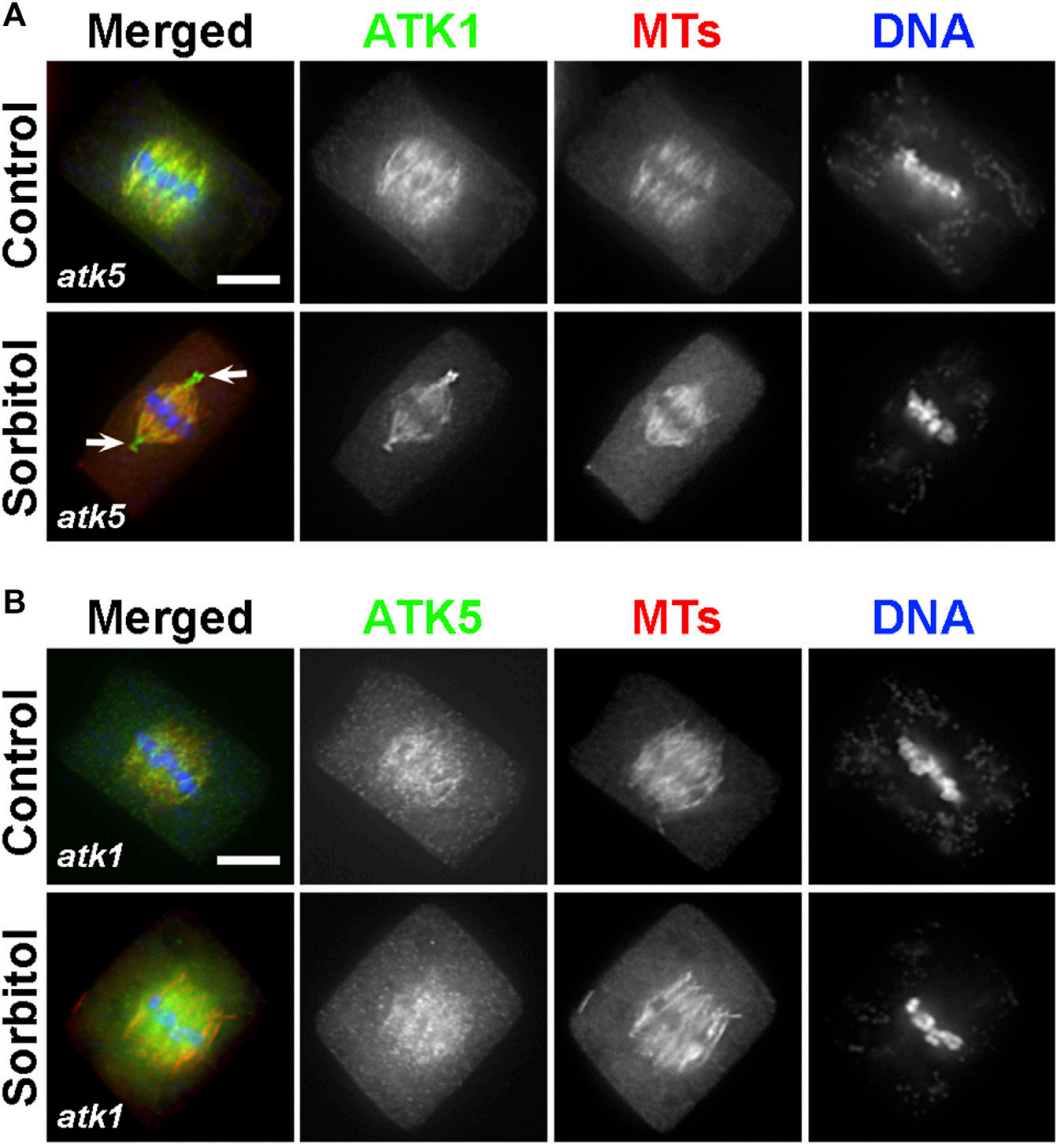
ATK1 and ATK5 associate with spindle MTs independently. **(A)** In the *atk5* background, ATK1-GFP localizes along spindle MTs and becomes highly enriched after sorbitol treatment at pseudo-centrosome foci that do not have obvious MT enrichment. **(B)** In the *atk1-1* background, ATK5-GFP still decorates spindle MTs but does not form pseudo-centrosomes after sorbitol treatment. Scale bars, 5 µm.

Because ATK1 and ATK5 shared similar localization patterns on spindle MTs, we asked whether there was a requirement for each other to achieve the spindle MT association. To do so, ATK1-GFP was expressed in the *atk5* mutant and ATK5-GFP was expressed in the *atk1* mutant. We found that neither ATK1-GFP localization on spindle MTs was affected by the *atk5* mutation, nor was ATK5-GFP localization affected by *atk1* (Figures 8A,B). Therefore, we concluded that the two Kinesin-14A motors achieve their spindle association independently from each other.

Because both *atk1* and *atk5* mutant cells formed mitotic spindles with disorganized poles, we then tested whether the sorbitol-induced pseudo-centrosomes might be formed independently by either ATK1 or ATK5. We found that in the *atk5* mutant, ATK1-GFP was concentrated at pseudo-centrosomes following sorbitol treatment at metaphase and the phenomenon was indistinguishable from that in the control cells (Figure 8A). In contrast, however, ATK5-GFP remained on spindle MTs in the *atk1* mutant background following sorbitol treatment (Figure 8B). Concomitantly, spindle poles remained wide in the *atk1-1* mutant under hyperosmotic conditions. Therefore, we concluded that ATK1 played a driving role in spindle pole-focusing and the formation of pseudo-centrosomes enriched with Kinesin-14A motors, and ATK5 was dependent on ATK1 for the redistribution.

### ATK1 Plays a Role in the Association of Augmin With Spindle MTs

**FIGURE 9:**
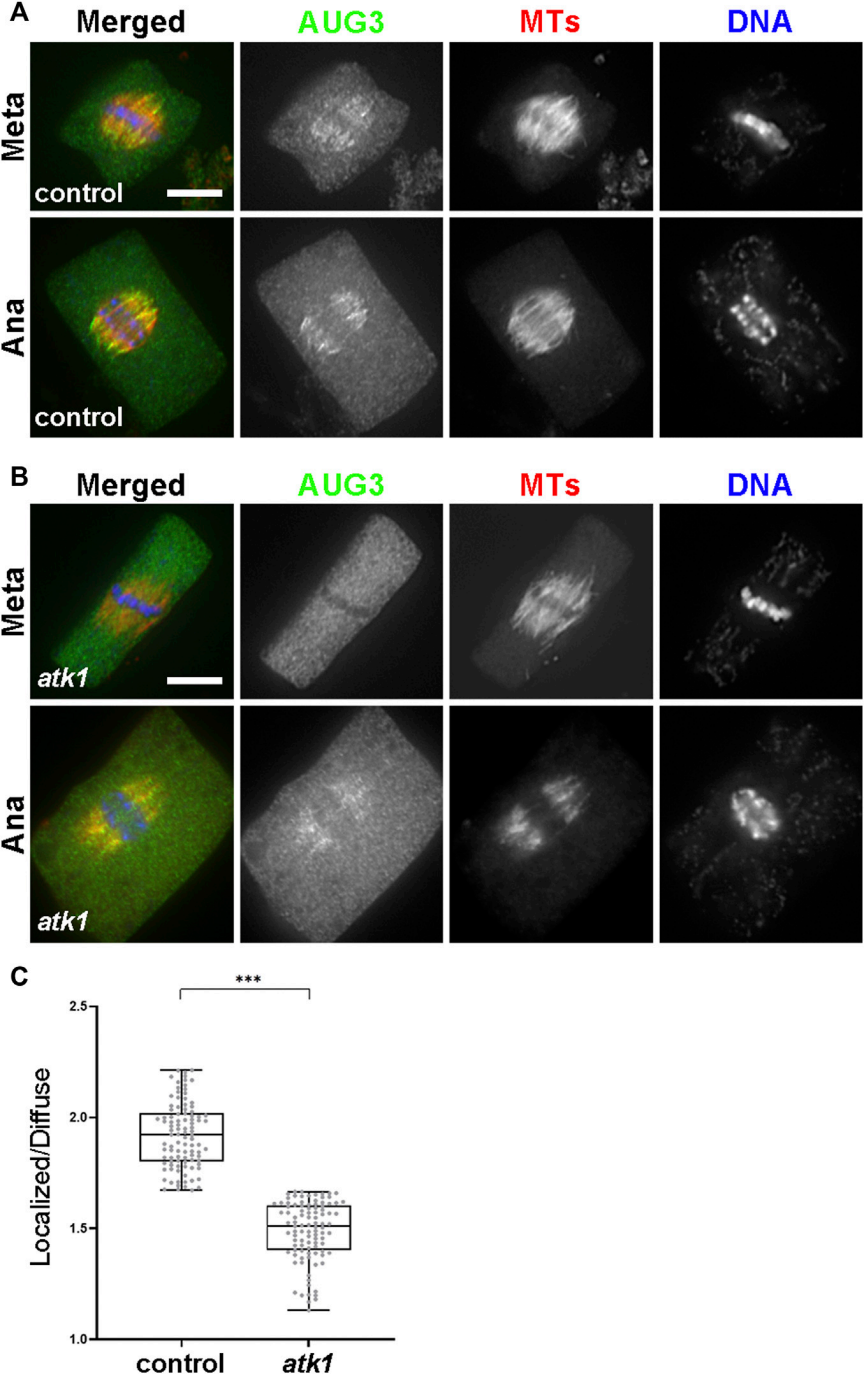
ATK1 plays a role in augmin association with MTs of the metaphase spindles. **(A)** Augmin subunit AUG3 shows an enriched localization on spindle MTs in both the metaphase (Meta) and anaphase (Ana) cells. **(B)** In the *atk1-1* mutant, however, AUG3 becomes noticeably weaker along spindle MTs when compared to the diffuse signal in the cytosol, which is particularly obvious in the metaphase cell. **(C)** Quantitative assessment of the MT-localized AUG3 signal, as reported by the ratio of spindle-associated vs. diffuse signals in metaphase cells. The ratios drop sharply in the *atk1-1* mutant. Scale bars, 5 µm.

Finally, we asked whether such joint action of ATK1 and ATK5 towards spindle poles, because of their essential contribution to spindle pole organization, was required for the association of MT nucleating factors with spindle MTs. Among these factors, the augmin complex plays a critical role in the spindle association of the γ-TuRC (Hotta, 2012). We expressed AUG3, an integral subunit of the augmin complex, in a GFP fusion in the *atk1-1* mutant. When compared to the control cells expressing the identical AUG3-GFP fusion which showed a concentrated association with spindle MTs (Figure 9A), the fusion protein exhibited a localization pattern with a more diffuse signal in the cytoplasm (Figure 9B). Such a phenomenon was particularly evident in metaphase cells in which the localized and diffuse signals were often indistinguishable in intensities (Figure 9B). Such a difference was significant when the MT-localized vs. diffuse signals were compared and quantified (Figure 9C). Therefore, we concluded that the ATK1-ATK5 oligomers play a critical role in the association of the MT nucleation factor augmin to spindle MTs for MT-dependent MT generation.

## Discussion

**FIGURE 10:**
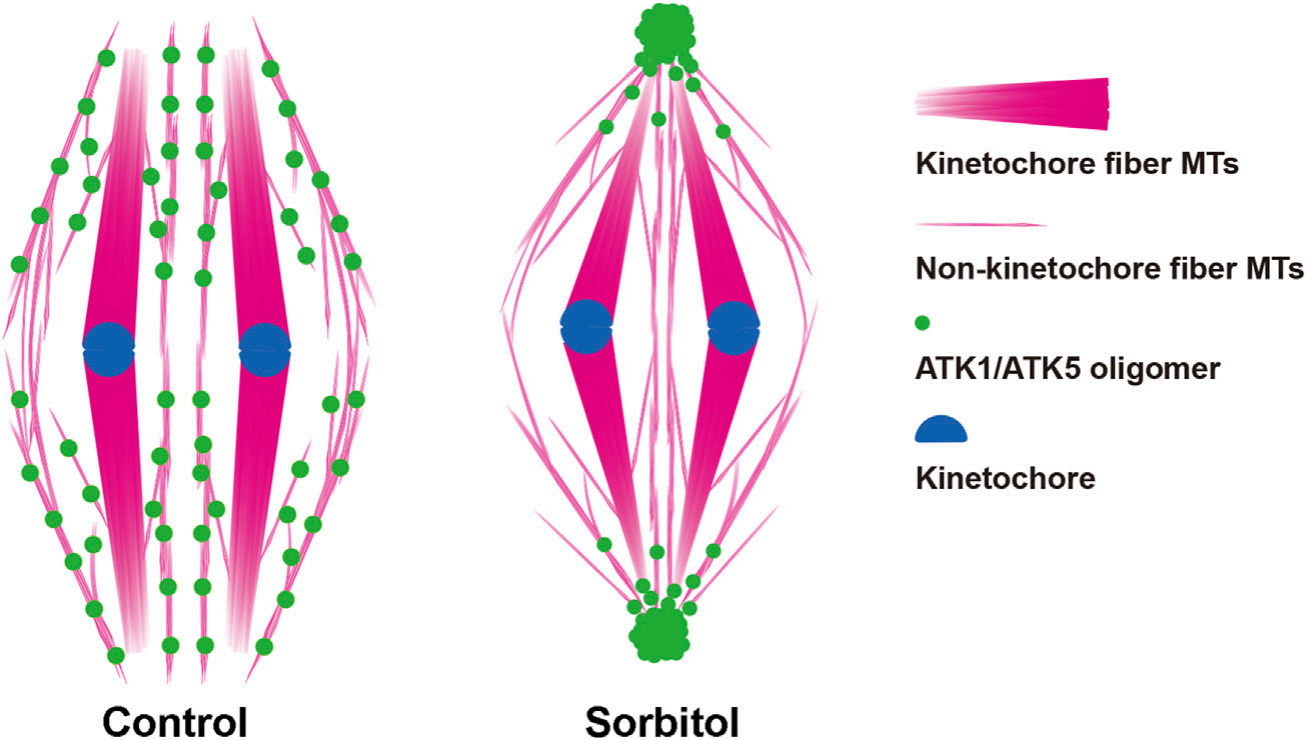
Diagrammatic representation of the redistribution of ATK1/ATK5 oligomers and reorganization of spindle MT arrays after sorbitol treatment.

It has been puzzling whether mitotic spindle MTs converged toward virtually unified poles in plant cells that lack the centrosome structure (Figure 10). Our results showed that the two highly homologous Kinesin-14A motors, ATK1 and ATK5 were physically associated with each other and exhibited incompletely redundant functions in converging spindle MTs toward unified poles. We found that these Kinesin-14A motors appeared in pseudo-centrosome foci upon hyperosmotic treatment (Figure 10), and these foci may be associated with pole convergence of mitotic spindles, as demonstrated here in *A*. *thaliana*.

### Spindle Pole Organization and Spindle Morphogenesis in Mitotic Plant Cells

Plants are known to produce what is often described as “barrel-shaped” spindles with great morphological plasticity that is often reflected by a wide range of pole width (Palevitz, 1993). In some specialized cells like the generative cell of the spiderwort *Tradescantia virginiana*, the mitotic spindle has kinetochore fibers interconnected that become unified together at anaphase in two “superbundles” belonging to two half spindles (Liu and Palevitz, 1992). In plant somatic cells, however, mitotic spindles have kinetochore fibers that are seemingly distance from each other when examined by anti-tubulin immunofluorescence or GFP-tagging fluorescence microscopy (Marcus et al., 2002). Such an organization pattern is not because plant cells lack necessary toolkit for driving focused MT organization or capability to do so. For example, MTs often are organized or centralized towards what is described as MT-converging centers in mitotic cells (Bajer et al., 1993). These MT-converging centers become polarized and evolved into two perfectly focused poles of spindles formed prior to nuclear envelop breakdown. These focused poles are enriched with MT nucleating factors like γ-tubulin, which is part of polar caps (Liu and Lee, 2022). The “loosening” of spindle poles is concomitant with the nuclear envelope breakdown and the dissolvement of polar caps. Nevertheless, spindle MTs always exhibit the pattern of convergence as if they are pointing towards virtually defined foci.

In acentrosomal spindles produced by plant cells, perhaps there are two major populations of MTs after chromosomes are attached to kinetochore fibers (Figure 10). In addition to these highly bundled kinetochore fibers, those non-kinetochore fiber MTs were placed between kinetochore fibers although being less noticeable by fluorescence microscopy. Although they probably did not run continuously from one spindle pole to the other as the interpolar MTs in animal centrosomal spindles, there are suggested here to play a critical role in spindle morphogenesis. Therefore, we took them as interpolar MTs here for their function in the convergence of kinetochore fibers towards poles, similarly to their animal counterparts that are linked to oppositely positioned centrosomes. It is unclear how these interpolar MTs were orchestrated to converge toward spindle poles when there were no physical anchoring points like the centrosomes.

In mitotic cells of animals, the spindle MT converging pattern is thought to be brought about by sliding of parallel MTs towards spindle poles *via* MT minus end-directed motors of cytoplasmic dynein and an evolutionarily conserved, ATK1/5-like Kinesin-14 like the human HSET protein (Borgal and Wakefield, 2018). Such a MT sliding activity becomes even more critical for mitosis in the absence of the centrosome. In acentrosomal cancer cells, for example, HSET is essential for organizing MTs into bipolar spindles during mitosis (Kleylein-Sohn et al., 2012). These animal acentrosomal spindles resemble those in plant cells. In the endosperm of the flowering plant *Haemanthus*, the observed formation of the MT-converging centers is established independent of chromosomes (Smirnova and Bajer, 1998), and is likely a result of MT sliding driven by one or more Kinesin-14 motors, simply because plants lack cytoplasmic dynein. Despite the functional redundancy among Kinesin-14 motors, as a result of the expansion of the Kinesin-14 genes in land plants, the loss of a single Kinesin-14A motor can lead to defects in spindle assembly as revealed during male meiosis and endosperm mitosis (Chen et al., 2002; Higgins et al., 2016; Huang et al., 2019). Similar phenotypes in spindle morphogenesis linked to mutations in comparable Kinesin-14A motors in both *A*. *thaliana* and maize suggest a conserved role in spindle pole organization. This phenotype may be caused by the lack of sufficient MT sliding in these selective cells in which other redundant motors may not be always expressed at a significant level. This notion is supported by the incomplete penetrance of the phenotypes in spindle assembly as the mutations can always be transmitted through gametes that are produced by mitosis during gametogenesis. In *A*. *thaliana*, inactivation of either *ATK1* or *ATK5* would result in disorganization of spindle poles in somatic mitotic cells albeit not noticeably sabotaging mitotic progression (Marcus et al., 2002; Marcus et al., 2003; Ambrose et al., 2005). The synthetic lethality of *atk1-1* and *atk5* suggests that the complete loss of these two redundant Kinesin-14 motors results in defects in spindle assembly that become too severe to be transmitted (Quan et al., 2008). A conclusion of such a function can be drawn after learning the consequence of perhaps conditionally inactivation of both ATK1 and ATK5 motors simultaneously and observing spindle assembly phenotypes. Another intriguing question is how these two nearly identical motors acquire certain unshared functions, *e*.*g*., during male meiosis while serving similar fundamental roles in spindle morphogenesis.

### ATK1-ATK5 Interaction for Spindle Pole Organization

MT sliding in spindle pole organization requires processive actions of MT minus end-directed motors like cytoplasmic dynein (Borgal and Wakefield, 2018). In order to do so, it was hypothesized that motors would form oligomers in order to achieve long-distance motility (Wittmann et al., 2001). Unlike cytoplasmic dynein, the Kar3p/NCD/HSET-typed Kinesin-14 motors are known to be non-processive motors, as demonstrated by ATK1 *in vitro* (Marcus et al., 2002). Therefore, such Kinesin-14 motors could acquire processive motility by oligomerization in order to function in MT sliding, an activity required for MT convergence in spindle morphogenesis.

Here, we discovered that ATK1 and ATK5 contribute to MT sliding during spindle assembly by direct association that was supported by three lines of evidence. First, our reciprocal purification results indicated that the two motors are associated with each other *in vivo*. This association likely took place between homodimers of the motors to form oligomers instead of forming ATK1-ATK5 heterodimers. This statement was supported by the fact that the two motors were spatially separated prior to nuclear envelope breakdown, with ATK1 in the nucleus and ATK5 in the cytosol. Moreover, ATK5 was associated with MTs in the prophase spindle where ATK1 was not detected. The second evidence came from the reconstitution of the GFP fluorescence resulting from the association of sfGFP^1-10^ with sfGFP^11^ fragments separately fused with ATK1 and ATK5 and expressed under their native promoters. Because the fluorescence was not reconstituted when the two fragments were fused with ATK1 and AUG3, we concluded that the reconstitution only took place when two fusion partners interact with each other but not simply by self-association of the two sfGFP fragments. The third evidence came from the enhanced coalescence of ATK1 and ATK5 signals when cells were under hyperosmotic stresses. The formation of the “pseudo-centrosome” of ATK1 and ATK5 at spindle poles marked minus end-directed hyperactivation of the two motors, through processive motility on interpolar spindle MTs.

Different from the dependence of spindle pole organization on ATK1-ATK5 association-, the human HSET motor contributes to the minus end organization of microtubules into asters through the formation of HSET-tubulin clusters (Norris et al., 2018). The HSET-tubulin clusters MT minus end-directed processive motility which is probably required for aster formation. It was unclear whether the pseudo-centrosomes formed by ATK1 and ATK5 in sorbitol-treated cells required the contribution of tubulins. However, our results showed clearly that tubulins were not enriched there when we performed anti-tubulin immunofluorescence.

Nevertheless, oligomerization of Kinesin-14 described here may be just one example of spindle-associated motor ensembles that are suggested to allow the generation of complex behaviors during mitosis (Gicking et al., 2018).

Concomitant with the poleward accumulation of ATK1 and ATK5 upon the hyperosmotic treatment, the MT nucleator γ-tubulin also showed a more biased localization pattern towards spindle poles than in the control cells, although not as dramatic as ATK1 or ATK5. Therefore, it is unlikely that the motor oligomers are directly associated with the γ-TuRC in the spindle. Coincidently, minus end-directed, motor-driven movement of the γ-TuRC was detected in human mitotic spindles (Lecland and Lüders, 2014). Deciphering the interactomes of the γ-TuRC and ATK1/ATK5 probably would uncover how poleward accumulation of the γ-TuRC may be achieved.

A remaining question is how the association of ATK1 and ATK5 is established. Perhaps these motors first formed homodimers followed by the lateral association of these dimers to form larger oligomers in a mitotically regulated manner. It will be interesting to find out whether the oligomerization is phospho-regulated by CDK/Cyclin and the mitotic kinase Aurora. Furthermore, do they form similar oligomers with other Kinesin-14A motors like KatB and KatC which, among Kinesin-14 motors in *A*. *thaliana* (Reddy and Day, 2001), show the highest sequence homology to ATK1 and ATK5?

### Kinetochore Fiber MTs and Interpolar MTs in the Spindle Assembly

Kinetochore fibers often are emphasized when spindle MTs are visualized by fluorescence microscopy, which has an inherited technical caveat of having weak signals of interpolar MTs shadowed by overwhelmingly bright signals of kinetochore fibers. In certain animal cells like the silkworm spermatocytes, however, centrosome-generated asters are significantly distanced from the spindle core of kinetochore fibers (Chen et al., 2008). In centrosomal spindles, MTs generated from segregated centrosomes give rise to interpolar MTs inside the spindle apparatus. Kinetochore fibers are attached laterally to these interpolar MT bundles (Tolic, 2018). Therefore, spindles have kinetochore fibers and interpolar MTs that likely harbor different molecular interactions simply because kinetochore fibers are attached to kinetochores while interpolar MTs are cross-linked near their plus ends by MAPs or motors. Obviously, it is challenging to tease the two populations of MTs apart because of their close spatial and/or possibly physical association (Tolic, 2018). However, when kinetochore fibers are removed from the spindle in grasshopper spermatocytes undergoing meiotic division, concomitantly with the surgical removal of chromosomes, interpolar MTs remain and can undergo cell cycle-dependent reorganization until the completion of cytokinesis (Zhang and Nicklas, 1996). This finding, although in meiotic cells, suggests that interpolar MTs can autonomously undergo M phase-dependent MT reorganization so that they likely play critical roles in spindle morphogenesis.

As shown here, the polar accumulation of ATK1 and ATK5 upon hyperosmotic stresses likely have interpolar MTs distanced from kinetochore fibers that were resulted from the sliding of primarily these MTs only. The resulting asters are similar to those generated by the centrosomes in spindles formed in silkworm spermatocytes (Chen et al., 2008). Kinetochore fibers remained in position probably because of the attachment to chromosomes in these sorbitol-treated cells. Our results indicated that ATK5 acted on interpolar MTs from late prophase prior to nuclear envelope breakdown and ATK1 join with ATK5 to these MTs afterward. The joint action of ATK1 and ATK5 is critical for the morphogenesis of spindles with converged poles as the loss of either one would cause defects in spindle pole convergence (Marcus et al., 2003; Ambrose et al., 2005). Our finding also further supports the notion that interpolar MTs are responsible for spindle morphogenesis.

In conclusion, we demonstrated here that the conserved ATK1 and ATK5 kinesins physically associated with each other and acted on selective parallel MTs in the mitotic apparatus to establish the convergent spindle array by MT sliding.

## Data Availability

The original contributions presented in the study are included in the article/Supplementary Material; further inquiries can be directed to the corresponding author.
