# Supplementary material for: Two Kinesin-14A Motors Oligomerize to Drive Poleward Microtubule Convergence for Acentrosomal Spindle Morphogenesis in Arabidopsis thaliana
Source: Front Cell Dev Biol. 2022 Jul 22;10:949345. doi: 10.3389/fcell.2022.949345 (PMC9380777; doi:10.3389/fcell.2022.949345)
Supplement: Supplementary file 2 [file Presentation1.PPTX]

## Slide 1
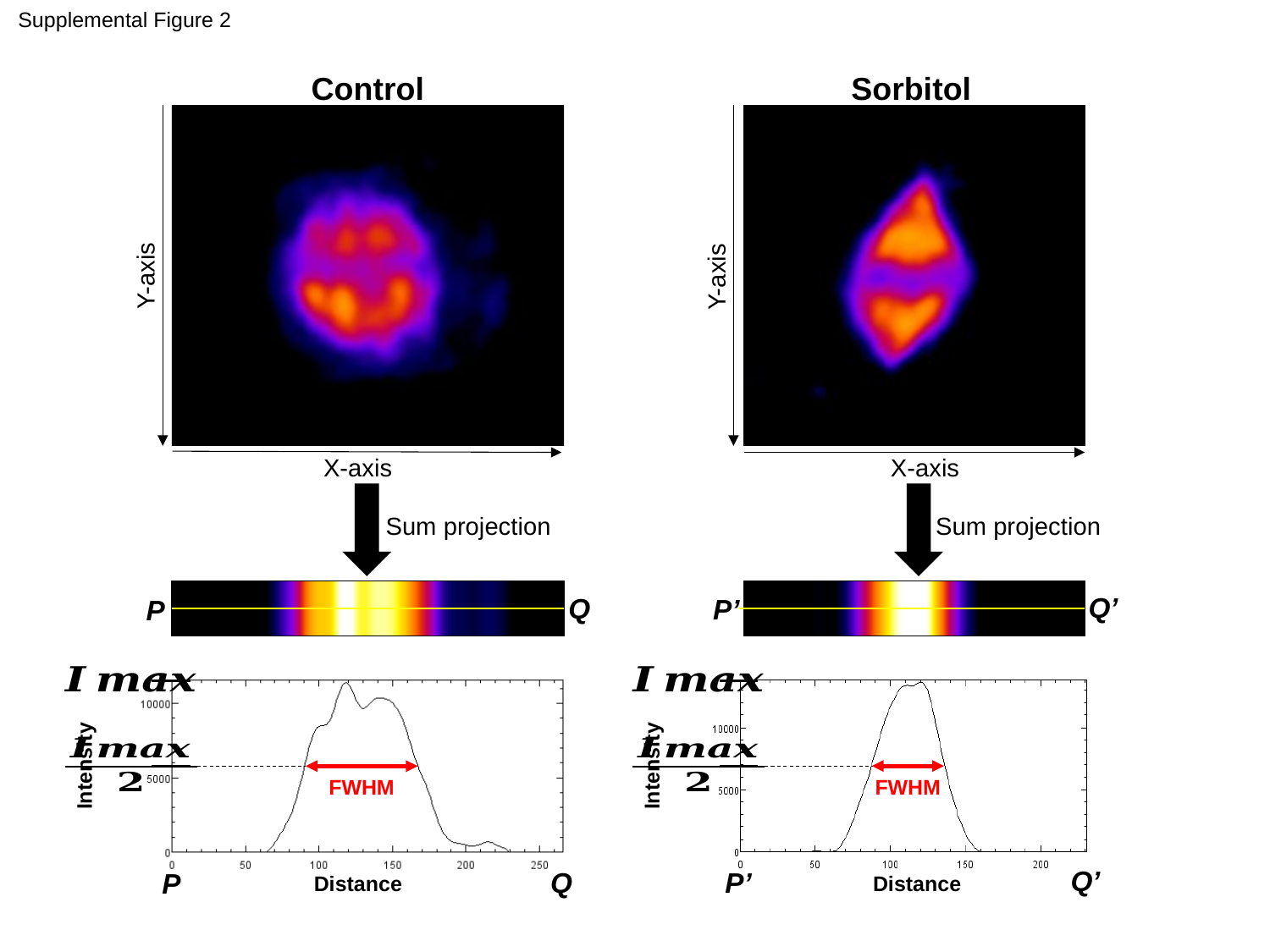

Supplemental Figure 2
Control
Sorbitol
Y-axis
Y-axis
X-axis
X-axis
Sum projection
Sum projection
Q’
Q
P’
P
Intensity
Intensity
FWHM
FWHM
Q’
Q
P’
P
Distance
Distance
